# Epigenetic age acceleration and neurotrophin signaling pathways in cancer-related cognitive impairment: a longitudinal, prospective cohort study

**DOI:** 10.3389/fragi.2025.1667638

**Published:** 2025-12-01

**Authors:** Michael Sayer, Ding Quan Ng, Julia Trudeau, Raymond J. Chan, Munjal M. Acharya, Kord Kober, Alexandre Chan

**Affiliations:** 1 School of Pharmacy and Pharmaceutical Sciences, University of California Irvine, Irvine, CA, United States; 2 College of Nursing and Health Sciences, Flinders University, Adelaide, SA, Australia; 3 School of Medicine, University of California Irvine, Irvine, CA, United States; 4 School of Nursing, University of California San Francisco, San Francisco, CA, United States; 5 Department of Oncology Pharmacy, National Cancer Centre Singapore, Singapore, Singapore

**Keywords:** cancer related cognitive impairment, epigenetic ageing, neurotrophin signaling pathways, brain derived neurotrophic factor, differential DNA methylation, pathway enrichment analysis

## Abstract

**Introduction:**

Biological age acceleration and disruptions in neurotrophin pathway signaling may significantly contribute to cancer-related cognitive impairment (CRCI) etiology. In this study, we evaluated the relationship of epigenetic age acceleration with cognitive function measures and circulating BDNF levels. Furthermore, we evaluated DNA methylation (DNAm) patterns to explore neurotrophin pathway associations with CRCI symptoms.

**Methods:**

In a longitudinal study, 51 newly diagnosed Adolescent and Young adult cancer patients and 8 age-matched healthy controls provided blood samples for DNAm and BDNF measurements with concurrent clinical assessments (#NCT03476070). We evaluated the relationship of epigenetic ageing with cancer status, circulating BDNF levels, and measured cognitive function. Next, we identified significant differentially methylated positions (DMPs), regions (DMRs), and significantly enriched pathways associated with BDNF and cognitive function outcomes.

**Results:**

PhenoAge and GrimAge demonstrated significant age acceleration relative to non-cancer controls and worsening cognitive function symptoms, with accelerated GrimAge associated with decreasing BDNF levels. DMPs associated with 5 different cognitive function outcomes (FactCog Score, Response, Memory, Executive Function, Multi-Tasking) were mapped to genes within KEGG pathway HSA:04722 (Neurotrophin Signaling Pathway). Key enriched pathways relative to both subjective cognitive function and multiple objective cognitive measurement domains were also enriched with respect to BDNF levels, including Synapse (GO:0045202), Glutamatergic Synapse (GO:0098978), and Neuron Projection (GO:0043005).

**Conclusion:**

Cancer and cancer treatment lead to significant epigenetic age acceleration, which can influence neuronal health and CRCI symptom onset. Furthermore, DNAm patterns corroborate BDNF as a potential biomarker for CRCI and suggest neurotrophin pathways play a meaningful role in CRCI etiology.

## Introduction

Cancer related cognitive impairment (CRCI), commonly known as “Chemobrain” or “Chemofog”, is characterized by impairment of memory, alertness or attention, learning, processing speed, or executive function (Ng et al., 2023). Its underlying causes are multifaceted, including biological stressors (inflammation, immune response, oxidative stress) and concurrent symptom burden (anxiety, depression, fatigue) (Cheng et al., 2024; Wang et al., 2022; Torre et al., 2021; Luo et al., 2023; Gullett et al., 2019). Discovering biomarkers for CRCI and evaluating its mechanistic causes is challenging. Systemic observations are difficult to generalize to the environment past the blood-brain barrier. Furthermore, cognitive deficits are difficult to attribute to cancer-specific causes when ageing and concurrent symptom burden also significantly influence cognition (Duivon et al., 2024; Demos-Davies et al., 2022).

Epigenetic measurements, like DNA methylation (DNAm), have the potential to advance CRCI research. Cancer and cancer treatment can significantly alter the epigenome, leading phenotypic changes which cause dysregulation of systemic processes and eventually lead to unwanted symptoms (Robinson et al., 2022; Wang and Zhang, 2025; Gibney and Nolan, 2010). DNA methylation measurements have the potential to be an indicator of biological stress caused by cancer. Epigenetic ageing measures may demonstrate this potential, as researchers have demonstrated biological age acceleration in cancer survivors, and that accelerated ageing leads to poorer health outcomes (Sayer et al., 2024; Wang et al., 2024; Sedrak et al., 2021). Various physiological changes associated with ageing have proven associations with cognitive function, potentially making them a useful biomarker tool in CRCI research (Lee and Kim, 2022).

Considering biomarker identification for CRCI specific applications, identifying key DNAm patterns that represent neuronal health and resilience could be impactful. Circulating brain-derived neurotrophic factor (BDNF) has been established as a surrogate for neurotrophin activity, which supports neuroplasticity needed for resilience to harmful exposures (Miranda et al., 2019; Bathina and Das, 2015). Preliminary evidence even suggests elevated BDNF levels decrease the likelihood of experiencing CRCI (Ng et al., 2023; Ng et al., 2017). DNAm patterns associated with circulating BDNF levels may be a surrogate of neuronal health, and eventually be utilized as a refined biomarker for CRCI care. Demonstrating significant DNAm relationships relative to cognitive function outcomes are associated with neurotophin activity are a needed first step.

This study harnesses epigenetic measurements with concurrent clinical assessments from a longitudinal, prospective cohort study featuring adolescent and young adult (AYA) cancer patients with non-cancer controls. DNAm methylation measurements are utilized to advance biomarker research for CRCI, and gain insights into its mechanistic causes. First, we evaluated associations of accelerated epigenetic ageing with cancer status and treatment, circulating BDNF levels, and cognitive function. Furthermore, we explored differential methylation patterns relative to cognitive function measures and circulating BDNF to assess the extent they support the role of neurotrophin pathways in CRCI etiology.

## Methods

### Study design and participants

Our study utilizes blood samples and data collected from a prospective, longitudinal, observational study following adolescent and young adult (AYA) cancer patients and non-cancer controls over the course of a year conducted at three ambulatory care centers in Singapore between June 2018 and June 2022 (CIRB 2017/3139, Clinicaltrials.gov: NCT03476070) (Ng et al., 2023; Chan et al., 2023). All research was performed in accordance with the Declaration of Helsinki and relevant institutional guidelines/regulations for human subject research, and all participants and/or legal guardians provided written informed consent prior to participation.

Recruited cancer patients were between 15 and 39 years old, newly diagnosed, treatment naïve, and able to provide informed consent (with parental consent if needed). Exclusions included evidence of psychosis or neuropsychiatric illness impairing cognitive abilities. *Non-cancer controls* had similar eligibility criteria excluding cancer diagnosis. Cancer patients were evaluated up to 5 times in 3-month intervals (Timepoints 1-5), beginning prior to anti-cancer therapy. Non-cancer controls were evaluated twice, once at baseline and once 6 months after baseline. Sample collection for non-cancer controls was limited; The underlying study design assuming the adolescent/young adult non-cancer population would not experience nuanced changes in clinical status or biomarker measurements over the course of a year. Data utilized for this study come from the larger trial previously mentioned, with this evaluation only considering entries with accompanying DNAm measurements. Data were collected through interviews and medical records, and participants completed tests, questionnaires, and blood draws administered by trained personnel.

### Data collection and management

#### Clinical symptom measurements

Objective Cognitive function was characterized with Cambridge Neuropsychological Test Automated Battery (CANTAB) tests, evaluating cognitive domains of memory, response speed, executive function, attention, and multi-tasking (Cacciamani et al., 2018). Reliable change indices (RCI) were determined specific to each domain relative to timepoint 1 assessments and utilized as outcomes, with lower scores indicating worsening performance relative to baseline. An outcome of “Objective cognitive impairment” was characterized as an observed RCI < −1.96 for any domain at a given measurement time (Jaco et al., 1991). Subjective cognitive function was captured with The Functional Assessment of Cancer Therapy-Cognitive Function version 3 (FACT-Cog), utilizing summed scores from survey responses as an outcome, with lower scores indicating worsening cognitive function (Cheung et al., 2013). An outcome of “Clinically significant subjective cognitive impairment” was defined based on an established minimal clinically importance difference threshold, which is a ≥10.6-point decline in Fact-Cog Scores relative to baseline assessment (Cheung et al., 2014).

The Multidimensional Fatigue Symptom Inventory-Short Form (MFSI-SF) and the psychological distress domain of Rotterdam Symptom Checklist (RSCL-PD) captured concurrent fatigue symptoms and psychological distress (Chan et al., 2018a; Chan et al., 2018b). Scores for each set of survey responses were summed and subsequently utilized, with higher scores representing worsening symptoms. More in-depth discussions of clinical symptoms measurement tools can be found in Supplementary Section 1.

#### Concurrent data

Additional treatment data included exposures to distinct classes of chemotherapy agents (platinum compounds, taxanes, doxorubicin) and radiation therapy (binary variables). Data from timepoints prior to chemotherapy initiation were labeled as “prior” samples, those taken prior to or within 1 month of a patients last chemotherapy dose were labeled “Active Therapy”, those >1 but <6 months removed were characterized as “Recent Therapy”, and those 6+ months removed from their last chemotherapy dose were labeled as “Stable”. Demographic data considered included the patient’s age, sex, ethnicity, and education status. Lastly, sample collection timing was characterized as the number of days removed from trial initiation for the timepoint.

#### DNA methylation measurements

DNA methylation measurements were performed on buffy coat samples collected from blood draws, utilizing the Illumina Infinium^®^ Methylation EPIC Array platform. For analysis, six different epigenetic age measures were derived from the data including Horvath, Hannum, PhenoAge, Horvath Skin and Blood (Horvath2), GrimAge, and DunedinPace (Horvath, 2013; Hannum et al., 2013; Levine et al., 2018; Horvath et al., 2018; Lu et al., 2019; Belsky et al., 2022). Methylation measurements at individual sites were utilized as M-values for subsequent analyses evaluating epigenetic associations, considering sites meeting quality control measures and those having meaningful biological variation. To correct for lingering technical variation and potential batch effects, principal components derived from M-Values were utilized (Price and Robinson, 2018).

In depth DNA methylation data management and methods can be found in the Supplementary Section 2.

#### BDNF measurements

Measurement of brain derived neurotrophic factor (BDNF) for this study has been described for samples included in this trial previously (Ng et al., 2023). Plasma BDNF was quantified utilizing enzyme-linked immunosorbent assay (ELISA) kits (Biosensis BEK-2211-1P/2P, Australia), and reported as concentrations of ng/mL. Measured BDNF concentrations were normalized through log-transformation prior to utilization in analyses.

### Analysis plan

#### Linear mixed modeling approach

To account for repeated measures, linear mixed models were applied in several different analyses. Covariates for multivariable models were selected from univariable evaluations, selecting features with p-values <0.1. Next, multivariable models including predictor(s) of interest and selected covariates were utilized. Wald statistics were used to assess feature significance (significance threshold: p < 0.05).

#### Association of epigenetic ageing with cancer and cancer treatment

Utilizing each epigenetic age as separate outcomes, linear mixed models were implemented with cancer treatment status as the primary predictor of interest. The cancer treatment status variable characterized samples into 5 categories, 1 for non-cancer controls (reference group), and the rest reflecting treatment trajectory for cancer patients (prior to therapy, active therapy, recent therapy, and stable). This analysis was repeated only including cancer patient samples (reference group = prior to therapy measurements).

#### Association of epigenetic ageing with circulating BDNF levels

To assess the relationship of epigenetic age acceleration with circulating BDNF levels, linear mixed models were implemented. Circulating BDNF levels were the outcome of interest, and epigenetic ageing measures were considered predictors of interest in separate analyses.

#### Association of epigenetic ageing with cognitive function in cancer patients

Cognitive function outcomes (FactCog Score and 5 RCI measures for CANTAB domains) were utilized as separate outcomes in linear mixed models, with each epigenetic age measure considered separately as primary predictors of interest. Samples included were from cancer patients at timepoints 2 through 5. Similar processes were followed utilizing clinically significant objective and subjective impairment as binary outcomes, except mixed effects logistic regression models were implemented.

#### Identification of differentially methylated positions

Differentially methylated positions (DMPs) were identified relative to each cognitive function outcome, using linear mixed models. The primary predictors of interest were M-values for each methylation site, evaluated independently. Cancer patient samples from timepoints 2 though 5 were included in these evaluations. Differentially methylated positions relative to BDNF levels were identified, utilizing circulating BDNF levels as an outcome and samples from cancer patients timepoints 1 to 5. Benjamini-Hochberg (BH) corrected p-values (q-values) were utilized to assess significance of methylation sites, utilizing a threshold of q < 0.2 (Ehlinger et al., 2023; Zhou et al., 2011; Montano et al., 2016).

#### Identification of differentially methylated regions

Utilizing Wald statistic p-values associated with methylation sites from DMP evaluations along with genomic coordinates, differentially methylated regions (DMRs) were identified utilizing “combp” algorithm (Jia et al., 2021). Significant DMRs had corrected p-values less 0.05, 3 or more DNAm sites associated with the region, and consistent directionality with respect to regression coefficients. Genomic coordinates and gene associations with DMPs and DMRs were based on probe-to-gene mapping from the Illumina Methylation EPIC array manifest, which utilizes HGNC-approved gene symbols (Hansen, 2016).

#### Identification of significantly enriched gene ontology pathways

For gene set enrichment analysis (GSEA), methylation sites meeting the suggestive significance threshold (q < 0.2) relative to each outcome were included. Pathway analyses considered terms and pathways ranging from 10–2000 genes within Kyoto Encyclopedia of Genes and Genome (KEGG) and Gene Ontology (GO) libraries utilizing MissMethyl software package in the R platform (Phipson et al., 2016). Enrichment significance was assessed based on hypergeometric tests, with a significance threshold of q < 0.2 based on Benjamini-Hochberg (BH) corrected p-values (q-values).

#### Overlap of significant differentially methylated positions, differentially methylated regions and enriched pathways

Overlapping DMPs, DMRs, and enriched pathways were evaluated relative to each cognitive function outcome. We defined “Overlapped” as an observed significant DMP, DMR, or enriched pathways occurring relative to FACT-Cog Score and 2 or more Objective Cognitive function outcomes. After discovering key overlaps related to cognitive function outcomes, we assessed how many of them were relevant to significant findings relative to BDNF.

#### Emphasizing findings relevant to neurotrophin signaling and production

Utilizing the molecular signatures database, we identified GO pathways and KEGG terms associated with Neutrophin Signaling and brain derived neurotrophic factor. We queried term and pathway names using the keywords “Neurotrophin,” “Brain Derived Neurotrophic Factor,” and “BDNF.” Gene sets associated with each term were collected, and DMPs and DMRs mapped to genes within these pathways were identified. The molecular signatures database was queried, and gene sets were collected utilizing the “msigdbr” R-package (Dolgalev, 2025).

Supplementary Section 3 provides detailed descriptions of statistical and bioinformatics methods implemented.

## Results

### Patient population and sample collection

In total, 51 AYAC patients and 8 non-cancer controls were recruited for the study. Mean age was slightly higher amongst AYAC patients (32.5 years old AYAC vs. 28.1 HC), with most of both populations having received a college degree (AYAC: 58.8% and HC: 75%), being of Chinese descent (AYAC: 76.5% and HC: 62.5%), and female (AYAC:60.8% and 25%) (Table 1). Amongst all AYAC patients, 51% received radiation therapy (n = 26), with platinum compounds the most common chemotherapy exposure (66.7%, n = 34), followed by doxorubicin (27.5%, n = 14), and taxanes (25.5%, n = 13).

**TABLE 1:** Baseline Patient Characteristics and Samples Collected.

Characteristic	AYAC patients	Healthy matched controls
Demographics		
*Total patients*	n = 51	n = 8
*Mean (Median) age*	32.5 (34)	28.2 (31)
*Chinese descent*	76.5% (n = 39)	62.5% (n = 5)
*Graduate degree attained*	58.8% (n = 30)	75% (n = 6)
*Male sex*	39.2% (n = 20)	25% (n = 2)
Cancer therapy exposures		
*Radiation therapy*	51% (n = 26)	
*Doxorubicin*	27.5% (n = 14)	
*Platinum*	66.7% (n = 34)	
*Taxanes*	25.5% (n = 13)	
Samples collected		
*Total samples*	177	16
*Timepoint 1*	28.8% (n = 51)	50% (n = 8)
*Timepoint 2*	24.9% (n = 44)	0
*Timepoint 3*	15.8% (n = 28)	50% (n = 8)
*Timepoint 4*	14.1% (n = 25)	0
*Timepoint 5*	16.4% (n = 29)	0
*Active therapy*	22% (n = 39)	0
*Recent therapy*	21.5% (n = 38)	0
*Stable*	27.7% (n = 49)	0
Cognitive measures		
*Subjective impairment*	20.6% (n = 26%)	
*Mean (SD) FACT-Cog Score*	128.85 (19.4)	
*Objective impairment*	16.7%(n = 21)	
*Mean (SD) memory RCI*	0.06 (1.26)	
*Mean (SD) response RCI*	−0.39 (1.1)	
*Mean (SD) attention RCI*	0.27 (1.15)	
*Mean (SD) executive function RCI*	0.2 (1.01)	
*Mean (SD) multitask RCI*	0.61 (1.29)	

Characteristics related to adolescent/young adolescent cancer (AYAC) patients and matched controls are reported for demographics, cancer therapy exposures, and sample collection. Relevant descriptive statistics are utilized for numeric and categorical data. Categories not relevant to healthy controls are left blank.

AYAC patients provided 177 total samples spanning all 5 timepoints, and non-cancer controls provided 16 total samples at baseline and at 6 months. Collected samples amongst cancer patients were collected prior to therapy [28.8%, (n = 51)], during active therapy [22% (n = 39)], recently removed from therapy [21.5% (n = 38)], and 6+ months removed from therapy(Stable) [27.7% (n = 49)] (Table 1). Over the timeframe of the study, there were 21 instances of objective cognitive impairment (16.7% of collected outcomes) and 26 instances of subjective impairment (20.6% of collected outcomes), with numeric cognitive measures varying (Table 1).

#### Epigenetic ageing in cancer patients relative to non-cancer controls

**FIGURE 1:**
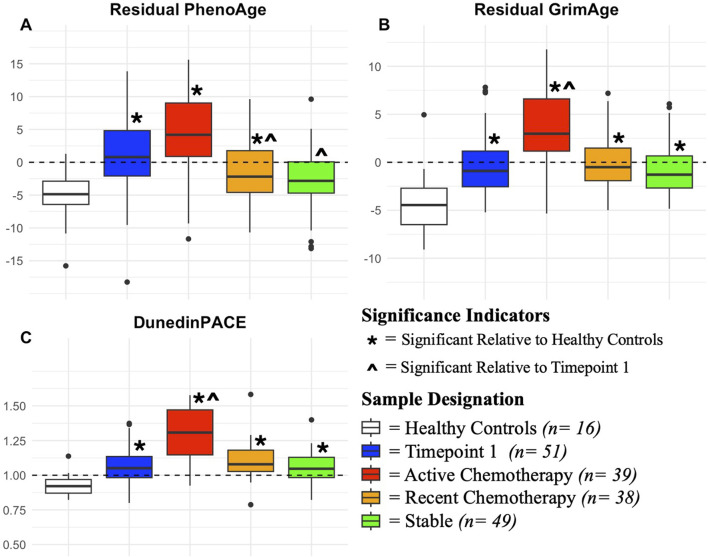
Trajectory of Epigenetic Ageing Relative to Cancer Treatment and Healthy Controls This figure contains boxplots demonstrating epigenetic ageing trends. Coloration of boxes represents distinct groups of patients based on cancer status and treatment trajectory. For each box, center lines represent median values, with boxes representing interquartile range, and tails (whiskers) being 1.5 times upper and lower quartile values. Y-axis values represent residual values for GrimAge and PhenoAge, with standard DunedinPACE measures utilized. Indicators (*) ( ^ ) demonstrate significance from regression analyses relative to indicated reference groupings (Wald-statistics, p < 0.05). Dotted lines represent neutral ageing values relative to each epigenetic ageing measure, with values above them suggesting accelerated ageing relative to expected. Each panel shows the trajectory a different epigenetic ageing measure, **(A)** residual PhenoAge, **(B)** residual GrimAge, and **(C)** DunedinPACE.

PhenoAge, GrimAge, and DunedinPACE were significantly elevated relative to healthy controls with timepoint 1 samples (PhenoAge Coef: 5.383, p < 0.001; GrimAge Coef: 3.61, p < 0.001; DunedinPACE Coef: 0.092, p = 0.023), active therapy samples(PhenoAge Coef: 8.42, p < 0.001; GrimAge Coef: 7.39, p < 0.001; DunedinPACE Coef: 0.317, p < 0.001), and recent therapy samples (PhenoAge Coef: 2.69, p = 0.045; GrimAge Coef: 4.63, p < 0.001; DunedinPACE Coef:0.144, p < 0.001). GrimAge and DunedinPACE were significantly elevated in stable measures relative to healthy controls as well (GrimAge Coef:4.54, p < 0.001; DunedinPACE Coef:0.124, p = 0.011) (Figure 1; Table 2; Supplementary Table S1).

**TABLE 2:** Relationship of Epigenetic Ageing Measures with Cancer Status, Treatment Status, Circulating BDNF Levels, and Cognitive Function Outcomes.

Overview	Interpretation	Horvath Age	Hannum Age	Pheno Age	Horvath2 Age	Grim Age	Dunedin PACE
Relationship of Accelerated Epigenetic Ageing with Cancer Status	Positive regression coefficients demonstrate accelerated epigenetic ageing in cancer patients relative to healthy controls within the designated treatment phase (reference value = Non-Cancer Controls)						
	Timepoint 1:	-0.435 (-2.96, 2.09) p= 0.73657	0.229 (-1.73, 2.19) p= 0.81901	5.383 (2.73, 8.03) p= 1e-04*	0.956 (-0.77, 2.69) p= 0.27979	3.605 (1.9, 5.31) p= 5e-05*	0.092 (0.01, 0.17) p= 0.02529*
	Active Treatment:	-1.38 (-3.95, 1.19) p= 0.29334	1.787 (-0.11, 3.69) p= 0.06703	8.416 (5.86, 10.97) p< 0.0001*	-0.531 (-2.29, 1.23) p= 0.55532	7.386 (5.72, 9.06) p< 0.0001*	0.317 (0.24, 0.4) p< 0.0001*
	Recent Treatment:	-1.812 (-4.43, 0.8) p= 0.17623	0.45 (-1.44, 2.34) p= 0.64044	2.686 (0.08, 5.29) p= 0.0448*	-1.237 (-3.02, 0.55) p= 0.17637	4.627 (2.97, 6.28) p< 0.0001*	0.144 (0.06, 0.22) p= 5e-04*
	Stable:	-0.883 (-3.43, 1.66) p= 0.49751	1.043 (-1.25, 3.34) p= 0.37345	1.441 (-1.7, 4.58) p= 0.36985	-0.634 (-2.36, 1.09) p= 0.47138	4.54 (2.53, 6.55) p= 2e-05*	0.124 (0.03, 0.22) p= 0.01197*
Changes in Epigenetic Ageing Across Cancer Patient Treatment Trajectory	Positive regression coefficients suggest accelerated epigenetic ageing relative to timepoint 1 measures amongst cancer patients (reference value= Timepoint 1)						
	Active Treatment:	-0.538 (-2.53, 1.45) p= 0.597	1.336 (-0.3, 2.97) p= 0.111	2.108 (-0.28, 4.5) p= 0.086	-1.23 (-2.52, 0.06) p= 0.064	3.841 (2.45, 5.23) p < 0.0001*	0.223 (0.16, 0.29) p< 0.0001*
	Recent Treatment:	-1.068 (-2.99, 0.85) p= 0.276	-0.232 (-2.16, 1.7) p= 0.814	-3.74 (-6.62, -0.86) p= 0.012*	-2.202 (-3.49, -0.92) p= 0.001*	1.363 (-0.23, 2.95) p= 0.095	0.072 (-0.01, 0.15) p= 0.07
	Stable:	-0.122 (-1.94, 1.7) p= 0.896	0.495 (-2.38, 3.37) p= 0.736	-5.332 (-9.52, -1.15) p= 0.014*	-1.643 (-2.84, -0.45) p= 0.008*	1.547 (-0.85, 3.95) p= 0.208	0.056 (-0.06, 0.17) p= 0.353
Relationship of Accelerated Epigenetic Ageing with Circulating BDNF Levels	Negative regression coefficients suggest as accelerated ageing increases, circulating BDNF levels decrease	0.029 (0, 0.05) p= 0.024*	0.017 (-0.02, 0.05) p= 0.341	-0.007 (-0.03, 0.02) p= 0.562	0.043 (0, 0.08) p= 0.028*	-0.041 (-0.08, 0) p= 0.028*	-0.717 (-1.48, 0.05) p= 0.067
Relationship of Accelerated Epigenetic Ageing With FactCog Score	Negative regression coefficients suggest as accelerated ageing increases, FactCog score decreases	0.157 (-0.4, 0.72) p= 0.583	-0.589 (-1.36, 0.19) p= 0.14	-0.557 (-1.01, -0.11) p= 0.017*	0.145 (-0.66, 0.95) p= 0.726	0.17 (-0.63, 0.97) p= 0.676	-4.823 (-21.55, 11.91) p= 0.573
Relationship of Accelerated Epigenetic Ageing with Subjective Cognitive Impairment Onset	Positive regression coefficients suggest as accelerated ageing increases, the likelihood of subjective cognitive impairment increases	0.016 (-0.11, 0.15) p= 0.811	0.198 (0.01, 0.38) p= 0.037*	0.022 (-0.08, 0.12) p= 0.675	0.171 (-0.02, 0.37) p= 0.084	-0.064 (-0.25, 0.12) p= 0.504	-1.217 (-4.78, 2.34) p= 0.503
Relationship of Accelerated Epigenetic Ageing With Memory RCI	Negative regression coefficients suggest as accelerated ageing increases, Memory RCI decreases	0.024 (-0.03, 0.08) p= 0.372	-0.082 (-0.15, -0.01) p= 0.021*	-0.044 (-0.08, -0.01) p= 0.024*	-0.026 (-0.1, 0.05) p= 0.516	-0.017 (-0.09, 0.05) p= 0.638	-1.126 (-2.52, 0.26) p= 0.116
Relationship of Accelerated Epigenetic Ageing with Response RCI	Negative regression coefficients suggest as accelerated ageing increases, Response RCI decreases	-0.004 (-0.05, 0.04) p= 0.855	0.017 (-0.04, 0.07) p= 0.532	-0.006 (-0.04, 0.02) p= 0.717	0.044 (-0.02, 0.11) p= 0.202	-0.041 (-0.09, 0.01) p= 0.119	-0.478 (-1.53, 0.58) p= 0.377
Relationship of Accelerated Epigenetic Ageing with Executive Function RCI	Negative regression coefficients suggest as accelerated ageing increases, Executive Function RCI decreases	0.014 (-0.02, 0.05) p= 0.474	0.027 (-0.02, 0.07) p= 0.256	0.006 (-0.02, 0.03) p= 0.632	0.009 (-0.05, 0.06) p= 0.758	0.028 (-0.02, 0.08) p= 0.278	0.284 (-0.67, 1.24) p= 0.561
Relationship of Accelerated Epigenetic Ageing with Multitask RCI	Negative regression coefficients suggest as accelerated ageing increases, Multitask RCI decreases	-0.003 (-0.05, 0.05) p= 0.898	0.028 (-0.04, 0.09) p= 0.388	-0.025 (-0.06, 0.01) p= 0.188	-0.029 (-0.11, 0.05) p= 0.461	-0.038 (-0.1, 0.03) p= 0.239	-0.387 (-1.72, 0.95) p= 0.571
Relationship of Accelerated Epigenetic Ageing with Attention RCI	Negative regression coefficients suggest as accelerated ageing increases, Attention RCI decreases	0.041 (0, 0.08) p= 0.062	0.01 (-0.05, 0.07) p= 0.737	-0.02 (-0.05, 0.01) p= 0.243	0.011 (-0.06, 0.08) p= 0.754	-0.062 (-0.12, 0) p= 0.05	-0.725 (-2, 0.54) p= 0.265
Relationship of Accelerated Epigenetic Ageing with Objective Cognitive Impairment Onset	Positive regression coefficients suggest as accelerated ageing increases, the likelihood of objective cognitive impairment increases	-0.019 (-0.12, 0.08) p= 0.723	0.096 (-0.03, 0.22) p= 0.146	0.079 (0, 0.15) p= 0.038*	0.02 (-0.14, 0.18) p= 0.805	0.145 (0.02, 0.27) p= 0.026*	1.981 (-0.54, 4.5) p= 0.124

This table represents results from several different analyses conducted utilizing epigenetic ageing metrics. The first column “Overview” is a brief statement describing the analysis. The “Description” describes the evaluation performed and the interpretation the reported of results based on expected outcomes. The next six columns each represent an epigenetic ageing measure, with reported results in each entry relative to the epigenetic age measure represented by the column titles. Results are regression coefficients, with accompanying 95% confidence intervals and p-values listed below. P-values less than 0.05 are indicated with asterisks (*) after the listed value

#### Epigenetic ageing amongst cancer patients

GrimAge and DunedinPace measures were significantly elevated in active therapy measures relative to Timepoint 1 measures (GrimAge Coef: 3.84, p < 0.001; DunedinPACE Coef: 0.223) (Figure 1; Supplementary Table S3). PhenoAge and Horvath2 were significantly lower in recent therapy (PhenoAge Coef: 3.74, p = 0.012; Horvath 2 Coef: −2.46, p < 0.001) and stable measures (PhenoAge Coef: 5.33 p = 0.014; Horvath 2 Coef: −1.73, p = 0.006) relative to timepoint 1 measures (Figure 1; Table 2; Supplementary Table S2).

#### Relationship of epigenetic ageing with circulating BDNF levels

Amongst epigenetic ages measures evaluated, accelerated GrimAge (Coef: 0.041, p = 0.028) was significantly associated with decreasing circulating BDNF levels (Table 2; Supplementary Table S3). Horvath (Coef:0.029, p = 0.024) and Horvath2 (Coef:0.043, p = 0.028) age accelerations were associated with increasing BDNF levels (Table 2; Supplementary Table S3).

#### Relationship of epigenetic age with cognitive function measures

Accelerated PhenoAge was significantly associated with decreasing FACT-Cog Scores (Coef: 0.557, p = 0.017) (Table 2; Supplementary Table S4). Additionally, accelerated Hannum Age was significantly associated with onset of subjective cognitive impairment (Coef:0.198, p = 0.037). For objective cognitive function outcomes, accelerated PhenoAge (Coef: 0.044, p = 0.024) and Hannum Age (Coef: 0.082, p = 0.021) were associated with decreased memory RCI (Table 2; Supplementary Table S4). Additionally, accelerated PhenoAge (Coef:0.079, p = 0.038) and GrimAge (Coef:0.145, p = 0.026) were significantly associated with onset of objective cognitive impairment (Table 2; Supplementary Table S4).

#### Identification of differentially methylated sites, regions, and enriched pathways relative to cognitive function outcomes

Evaluation of differentially methylated positions relative to FACT-Cog Score yielded 246 significant sites, with slight genomic inflation (入 = 1.35) (Supplementary Table S5). There were 9 differentially methylated regions (DMRs), and 43 enriched pathways (Supplementary Table S5). DMP analysis relative to objective cognitive function outcomes varied based on outcome assessed, with genomic inflation ranging from 0.81 to 1.32 (Supplementary Table S5). Memory RCI had the most significant DMPs (n = 1,592), DMRs (n = 28), and enriched pathways (n = 632). Attention and Executive Function RCIs did not have any enriched pathways due to limited DMPs eligible for inclusion for enrichment analysis (Supplementary Table S5).

No identified DMPs or DMRs meeting occurred in both FACT-Cog Score and 2 or more objective cognitive function domains assessed. Six pathways enriched with respect to FACT-Cog and 2 or more objective cognitive function outcomes (Table 3).

**TABLE 3:** Overlapping Enriched Pathways Relative to Cognitive Function Outcomes and Circulating Brain Derived Neurotrophic Factor.

Name	ID	Total pathways	Specific outcomes
Synapse	GO:0045202	4	Memory, FACT-Cog, Response, Multitask, **BDNF**
Collagen-containing extracellular matrix	GO:0062023	3	Memory, FACT-Cog, response
External encapsulating structure	GO:0030312	3	Memory, FACT-Cog, response
Extracellular matrix	GO:0031012	3	Memory, FACT-Cog, response
Glutamatergic synapse	GO:0098978	3	Memory, FACT-Cog, Multitask, **BDNF**
Neuron projection	GO:0043005	3	Memory, FACT-Cog, Response, **BDNF**

From left to right, the first column represents the pathway ID, followed by its name, the total number of outcomes had the pathway enriched, and specific outcomes overlapping. Pathways are listed if they were enriched relative to FACT-Cog score and 2 or more objective cognitive function measures; those enriched relative to BDNF have **BDNF** bolded and listed as well.

#### Identification of differentially methylated sites, regions, and enriched pathways relative to circulating brain derived neurotrophic factor levels

Evaluations relative to BDNF levels yielded 335 DMPS, 8 DMRs, and 29 enriched pathways (Supplementary Table S5). Amongst 29 enriched pathways idenfied, 11 referenced neuronal structures and neuronal signaling pathways (Supplementary Table S6). Amongst the 6 overlapping enriched pathways relative to cognitive function outcomes, 3 were also enriched relative to BDNF levels including Synapse (GO:0045202), Glutamatergic Synapse (GO:0098978), and Neuron Projection (GO:0043005) (Table 3).

#### Differentially methylated positions and regions mapped to genes within selected KEGG and GO terms

Query of the molecular signature data base identified 12 pathways (11 GO pathways, 1 KEGG term) associated with BDNF or neurotrophins. The KEGG term “neurotrophin signaling pathway” (HSA:04722) had the most overlapping genes mapped to significant DMPs and DMRs (Supplementary Table S7). Additionally, neurotrophin signaling pathway (GO:0038179), neurotrophin production (GO:0032898), and neurotrophin TRK receptor signaling pathway (GO:0048011) each had more than 1 DMP mapped to genes within them (Supplementary Table S7). Of the pathways assessed, three (HSA:04722, GO:0038179, GO:0032898) had significant DMPs relative to circulating BDNF levels. Cumulatively, 2 DMRs relative to Memory RCI and Executive Function RCI outcomes were mapped to genes within identified pathways (HSA:04722, GO:0038179, GO:0032898) (Supplementary Table S7).

## Discussion

We are the first study to observe accelerated ageing among AYA cancer patients receiving treatment over time, which influenced circulating BDNF levels and cognitive function measures. Observed DNAm patterns relative cognitive function outcomes emphasized genes, regions, and broader pathway enrichment trends that support the role of neurotrophin activity in the etiology of CRCI. In the future, DNAm-based biomarkers representative of neurotrophin activity and neuronal health may play a meaningful role in CRCI care. These markers demonstrate that accelerated aging contributes to the pathophysiology of CRCI, which provides important evidence that reversal of accelerated aging may help to curb the onset of CRCI in AYA cancer patients.

Our results highlighted that AYA cancer patients over the course of a year experience significant accelerated ageing relative to healthy controls, as well as during their cancer treatment. Rapid accelerated epigenetic ageing from treatment exposures have been suggested by other researchers, speculating that cancer and cancer treatment have acute effects on cell senescence pathways that modulate epigenetic patterns (Sehl et al., 2020; Xiao et al., 2021). Some have further suggested utilizing these measures to assess cumulative therapeutic toxicities experienced in cancer treatment, even including them cancer treatment clinical trials (Mittal and Vaughan, 2023). Others have started utilizing it as metric to demonstrate efficacy of intervention intended to treat survivorship issues, showing significant reductions over time with exercise and nutrition interventions (Hwang et al., 2022; Fitzgerald et al., 2021; Moulton et al., 2024).

Epigenetic ageing measures influenced by cancer treatment had expected associations BDNF levels, with accelerated ageing significantly associated with decreasing BDNF levels. Accelerated ageing measures may reflect physiological stressors that influence neurotrophin activity, further supporting them as biomarkers in CRCI care. In previous studies conducted by our team utilizing a larger subset of this patient data, we found increased BDNF levels to be significantly associated improved measured cognitive function and a decreased likelihood of CRCI (Ng et al., 2023; Trudeau et al., 2025). These findings suggest biomarkers reflecting cumulative systemic duress (inflammation, immune activity, oxidative stress) like biological ageing measures, used in conjunction with surrogates of neurotrophin activity may be ideal (Cribb et al., 2022; Kandlur et al., 2020).

Accelerated biological ageing, as represented by PhenoAge and GrimAge measures, also significantly predicted worsening cognitive measures and significant cognitive impairment. Our preliminary evidence along with results of other smaller studies, suggest meaningful associations with accelerated ageing and CRCI (Rentscher et al., 2023; Yang et al., 2020; Yang et al., 2022). Further studies have even demonstrated that Grimage and PhenoAge acceleration are associated clinically detrimental brain imaging measures (Hillary et al., 2021). Considering observed associations of these measures with cancer status and treatment trajectory, along with circulating BDNF levels and cognitive function measures, newer epigenetic clocks reflecting health status may have utility as biomarkers in survivorship care. However, specific DNAm markers relative to neuronal health and neurotrophin signaling may produce signals more specific to cognitive toxicities.

Differential methylation patterns relative to cognitive function outcomes implicated key genomic sites associated with neurotrophin signaling and production. For instance, for HSA:04722 (Neurotrophin Signaling Pathway) 5 different cognitive function outcomes assessed had differentially methylated positions mapped to 10 distinct genes within this pathway. Several are directly related to signaling from Trk receptor down-stream signaling cascades including PIK3CD (implicated genes: PIK3CD, GAB1, SH2B2, SH2B3) and MAPK/ERK (implicated genes: HRAS, RPS6KA2, RAP1A, SHC3, GAB1) based pathways (Kanehisa et al., 2013). The neuroprotective effects of BDNF and other neurotrophins are dependent on this pathway activity, beginning with binding these receptors (Park and Poo, 2013; Huang and Reichardt, 2003). Other gene ontology pathways emphasizing neurotophin signaling were not as well represented with identified DMPs and DMRs, which emphasized fewer genes directly related to up-stream TRK receptor molecular mechanisms as opposed to broader signaling cascades. However, our results specific to HSA:04722 do support a growing body of evidence that neurotrohpin activity and signaling play a meaningful role in CRCI symptoms and that potential interventions aimed to increase this pathway’s activity may provide symptomatic relief.

Significant DMPs and DMRs relative to FactCog Score and Memory RCI were mapped to genes within GO:0032898 (Neurotrophin Production) including NPY and ADORA1. In preliminary studies in animal models, signaling related to these genes are associated with upregulation of BDNF and broader neurotrophin production as a stress response (Zhang et al., 2020; Cohen et al., 2012). Additional clinical studies support the relationship of these gene products with psychiatric symptoms (Dong et al., 2022; van Calker et al., 2019). An additional differentially methylated position relative circulating BDNF levels was identified mapped to PCSK6 within GO:0032898 pathway. PCSK6 is responsible for converting precursor molecules into functioning BDNF messengers (Bogacheva et al., 2022). Further studies considering the relationship of precursor molecules and key enzymes responsible for neurotrophin formation with cognitive function may provide additional insights regarding CRCI etiology.

Broader pathway enrichment results, relative to cognitive function and BDNF, support BDNF’s neuroprotective role and its relationship with cognitive function. Of six overlapping enriched pathways relative to cognitive function outcomes, three including (synapse(GO:0045202), glutamatergic synapse (GO:0098978), and neuron projection (GO:0043005)) were also enriched relative to BDNF levels. This demonstrates cumulative epigenetic changes relative cognitive function measures had similarities to those influencing BDNF levels, further supporting its potential as a meaningful biomarker for CRCI. Researchers have previously created cumulative epigenetic measures the reflect dementia risk, physical activity levels, immune cell composition, protein biomarker levels, and risk of developing cancer (Jokai et al., 2023; Koetsier et al., 2024; Baron et al., 2018; Hillary and Marioni, 2020; Tanić et al., 2024). Emphasizing measures with data collected from cancer patients may lead to refined calibrate risk assessment tools specific to survivorship care.

Our results, suggesting epigenetic modifications within genes relevant to neurotrophin pathways play a role in CRCI, align with other studies that evaluate the mechanisms underlying CRCI. For instance, some have emphasized how systemic processes like inflammation and oxidative stress negatively impact the expression of plasma BDNF and other key neurotrophin signaling messengers, which leads diminished neuronal health and poorer cognitive function (Trudeau et al., 2025; Yap et al., 2021). Although others have suggested that elevated BDNF levels and increased neurotrophin pathway activity can create resilience to these destructive systemic processes, leading to exploration of interventions to increase neurotrophin pathway activity (Numakawa et al., 2011). The biological underpinnings of CRCI are complex and likely cannot be characterized evaluating neurotrophin pathways alone. Recent reports have suggested that mTOR pathways can play significant role in several biological processes influencing CRCI symptoms, including biological age acceleration and metabolic processes needed to maintain neuronal health (Fan et al., 2025; Zhao et al., 2024; Fan et al., 2022). Others have emphasized pathways related to synaptic vesicles and key neurotransmitters (i.e., dynamin-1) being influenced by cancer and cancer treatment, which may impact the onset and severity of CRCI (Nordhjem and Hjalgrim, 2025; Ng et al., 2025). Future studies should evaluate several different forms of omics data, along with epigenetic changes, in order to better capture the interplay of different pathways and CRCI etiology.

Given research demonstrating the association between BDNF and relevant biochemical/physiological pathways related to cognitive function, combined clinical studies demonstrating associations of circulating BDNF levels with the onset of cancer-related cognitive impairments, utilization of BDNF as an informative biomarker in clinical care seems like a logical next step. However, there are obstacles in translating circulating BDNF levels into actionable clinical insights. Assessment and diagnosis of CRCI remains challenging, with symptoms manifesting in a variety of different forms. Our team has previously reported that circulating BDNF levels had meaningful associations with objective cognitive tests, but that associations with self-reported cognitive function were mixed (Ng et al., 2022). Numerous factors aside from cancer and cancer treatment can influence BDNF levels (i.e., age, gender, physical activity), thus having expected “normal” values for stratified patient populations may be necessary (Miranda et al., 2019). Relying on change in BDNF values over time may be a more reliable approach as well, however rigorous sample collection strategies over time would be needed along with larger studies defining actionable “clinical significant” changes in circulating BDNF levels.

We do acknowledge that our study has limitations. While our heterogeneous patient population and sample collection are novel, generalization of our findings to specific cancer sub-types may be challenging. Having 51 patients with multiple measures is comparable to similar longitudinal studies and a significant accomplishment considering challenges recruiting AYA patients (Sayer et al., 2024; Docherty et al., 2019; Burke et al., 2007); However, follow-up evaluations in larger patient cohorts are warranted. This informed our analytical methodology, as evaluations regarding epigenetic aging measures were not subjected to multiple testing correction. Furthermore, significance thresholds were based on BH corrected thresholds of q less than 0.2; not more stringent thresholds were utilized elsewhere. Genomic inflation was observed specific to some DMP evaluations, however comparing results relative to several cognitive outcomes validated our significant findings.

## Conclusion

Cancer and cancer treatment lead to significant epigenetic age acceleration, which can influence neuronal health and CRCI symptom onset. Furthermore, DNAm patterns corroborate BDNF as a potential biomarker for CRCI and suggest neurotrophin pathways play a meaningful role in CRCI etiology. Our study also represents novel applications of DNAm data as a tool in biomarker discovery in CRCI research, and future studies should evaluate the association of various omics data with epigenetic changes, in order to better capture the interplay of different pathways and CRCI etiology.

## Data Availability

The raw data supporting the conclusions of this article will be made available by the authors, without undue reservation.

## Abbreviations

CRCI, Cancer Related Cognitive Impairment; DNAm, DNA Methylation; DMP, Differentially Methylation Position; DMR, Differentially Methylated Region; GO, Gene Ontology; BDNF, Brain Derived Neurotrophic Factor; AYA, Adult and Young Adult; CANTAB, Cambridge Neuropsychological Test Automated Battery; MFSI-SF, The Multidimensional Fatigue Symptom Inventory-Short Form; RSCL-PD, Rotterdam Symptom Checklist; ELISA, enzyme-linked immunosorbent assay.
