# Supplementary material for: Epigenetic age acceleration and neurotrophin signaling pathways in cancer-related cognitive impairment: a longitudinal, prospective cohort study
Source: Front Aging. 2025 Dec 1;6:1667638. doi: 10.3389/fragi.2025.1667638 (PMC12702849; doi:10.3389/fragi.2025.1667638)
Supplement: Supplementary file 1 [file Table1.docx]

**Supplemental File:**

**Supplemental Table 1:** **Comparison of Epigenetic Ageing Between Healthy Controls and Cancer Patients**

From left to right each column represents a different epigenetic ageing metric outcome. The first four rows are reported results specific to cancer status relative to healthy control samples, with rows below the black line representing univariable evaluations conducted to identify relevant confounders. At the bottom of the table, the selected confounders are listed for convenience. Each entry is reported as regression coefficients with corresponding 95% confidence intervals (Coefficient (95% CI)). P-values are reported beneath, with (*) indicators for values less than 0.05, and (^) symbols for values less than 0.1.

| **Feature** | **Horvath Age** | **Hannum Age** | **PhenoAge** | **Horvath2 Age** | **GrimAge** | **DunedinPACE** |
| --- | --- | --- | --- | --- | --- | --- |
| Timepoint 1  (ref= Healthy Controls) | -0.435 (-2.96, 2.09)  p= 0.73657 | 0.229 (-1.73, 2.19)  p= 0.81901 | 5.383 (2.73, 8.03)  p= 1e-04* | 0.956 (-0.77, 2.69)  p= 0.27979 | 3.605 (1.9, 5.31)  p= 5e-05* | 0.092 (0.01, 0.17)  p= 0.02529* |
| Active Chemotherapy  (ref= Healthy Controls) | -1.38 (-3.95, 1.19)  p= 0.29334 | 1.787 (-0.11, 3.69)  p= 0.06703 | 8.416 (5.86, 10.97)  p< 0.0001* | -0.531 (-2.29, 1.23)  p= 0.55532 | 7.386 (5.72, 9.06)  p< 0.0001* | 0.317 (0.24, 0.4)  p< 0.0001* |
| Recent Chemotherapy  (ref= Healthy Controls) | -1.812 (-4.43, 0.8)  p= 0.17623 | 0.45 (-1.44, 2.34)  p= 0.64044 | 2.686 (0.08, 5.29)  p= 0.0448* | -1.237 (-3.02, 0.55)  p= 0.17637 | 4.627 (2.97, 6.28)  p< 0.0001* | 0.144 (0.06, 0.22)  p= 5e-04* |
| Stable  (ref= Healthy Controls) | -0.883 (-3.43, 1.66)  p= 0.49751 | 1.043 (-1.25, 3.34)  p= 0.37345 | 1.441 (-1.7, 4.58)  p= 0.36985 | -0.634 (-2.36, 1.09)  p= 0.47138 | 4.54 (2.53, 6.55)  p= 2e-05* | 0.124 (0.03, 0.22)  p= 0.01197* |
|  |  |  |  |  |  |  |
| Chronological Age  (numeric) | 0.005 (-0.11, 0.12)  p= 0.93922 | 0.015 (-0.08, 0.11)  p= 0.75152 | 0.032 (-0.11, 0.17)  p= 0.66253 | 0.009 (-0.07, 0.09)  p= 0.82803 | 0.004 (-0.08, 0.09)  p= 0.92866 | -0.001 (-0.01, 0)  p= 0.49546 |
| Chinese Descent  (binary) | -1.779 (-3.38, -0.18)  p= 0.03086* | -0.854 (-2.09, 0.38)  p= 0.17669 | -1.468 (-3.4, 0.47)  p= 0.13886 | -0.783 (-1.84, 0.28)  p= 0.1502 | -0.332 (-1.48, 0.82)  p= 0.57151 | -0.016 (-0.07, 0.04)  p= 0.55132 |
| Male Sex  (binary) | 2.622 (1.26, 3.98)  p= 0.00021* | 1.704 (0.66, 2.75)  p= 0.0017* | -0.809 (-2.49, 0.88)  p= 0.34793 | 0.217 (-0.71, 1.14)  p= 0.64708 | 1.147 (0.16, 2.13)  p= 0.02346* | 0.011 (-0.03, 0.06)  p= 0.62164 |
| College Degree  (binary) | -1.231 (-2.62, 0.16)  p= 0.08448^ | -1.47 (-2.52, -0.42)  p= 0.0069* | -2.145 (-3.81, -0.48)  p= 0.01217* | -0.432 (-1.35, 0.49)  p= 0.35996 | -1.838 (-2.8, -0.88)  p= 0.00024* | -0.066 (-0.11, -0.02)  p= 0.00336* |
| Days Removed  (numeric) | 0 (0, 0)  p= 0.83623 | -0.005 (-0.01, 0)  p= 0.02133* | -0.01 (-0.02, 0)  p= 0.02027* | -0.002 (-0.01, 0)  p= 0.21783 | -0.006 (-0.01, 0)  p= 0.03954* | 0 (0, 0)  p= 0.01642* |
| PC1  (numeric) | 0.004 (0, 0.01)  p= 1e-05* | 0 (0, 0)  p= 0.85938 | -0.004 (-0.01, 0)  p= 0.0011* | 0.002 (0, 0)  p= 0.00011* | 0 (0, 0)  p= 0.97339 | 0 (0, 0)  p= 0.096^ |
| PC2  (numeric) | -0.003 (0, 0)  p= 0.02153* | 0.003 (0, 0)  p= 0.00049* | 0.005 (0, 0.01)  p= 0.00022* | 0 (0, 0)  p= 0.82785 | 0.003 (0, 0)  p= 0.00061* | 0 (0, 0)  p= 0.11776 |
| PC3  (numeric) | -0.001 (0, 0)  p= 0.5499 | -0.007 (-0.01, 0)  p< 0.0001* | -0.015 (-0.02, -0.01)  p< 0.0001* | -0.002 (0, 0)  p= 0.04787* | -0.006 (-0.01, 0)  p< 0.0001* | 0 (0, 0)  p= 0.00012* |
| PC4  (numeric) | -0.007 (-0.01, 0)  p= 0.00037* | -0.004 (-0.01, 0)  p= 0.01542* | -0.004 (-0.01, 0)  p= 0.0694^ | -0.002 (0, 0)  p= 0.07917^ | 0 (0, 0)  p= 0.79001 | 0 (0, 0)  p= 0.72568 |
| **Selected**  **Confounders** | Chinese Descent +  Male Sex +  College Degree +  PC1 +  PC2 +  PC4 | Male Sex +  College Degree +  Days Removed +  PC1 +  PC2 +  PC3 +  PC4 | College Degree +  Days Removed +  PC1 +  PC2 +  PC3 +  PC4 | PC1 +  PC3 +  PC4 | Male Sex +  College Degree +  Days Removed +  PC2 +  PC3 | College Degree +  Days Removed +  PC1 +  PC3 |

***PC- Principle component**

**Supplemental Table 2:** **Comparison of Epigenetic Ageing Between Across Different Phases of Cancer Treatment**

From left to right each column represents a different epigenetic ageing metric outcome. The first three rows are reported results specific to treat trajectory status relative to timepoint 1 samples, with rows below the black line representing univariable evaluations conducted to identify relevant confounders. At the bottom of the table, the selected confounders are listed for convenience. Each entry is reported as regression coefficients with corresponding 95% confidence intervals (Coefficient (95% CI)). P-values are reported beneath, with (*) indicators for values less than 0.05, and (^) symbols for values less than 0.1.

| **Feature** | **Horvath Age** | **Hannum Age** | **PhenoAge** | **Horvath2 Age** | **GrimAge** | **DunedinPACE** |
| --- | --- | --- | --- | --- | --- | --- |
| Active Chemotherapy (ref= Timepoint 1) | -0.538 (-2.53, 1.45)  p= 0.597 | 1.336 (-0.3, 2.97)  p= 0.111 | 2.108 (-0.28, 4.5)  p= 0.086 | -1.23 (-2.52, 0.06)  p= 0.064 | 3.841 (2.45, 5.23)  p< 0.0001* | 0.223 (0.16, 0.29)  p< 0.0001* |
| Recent Chemotherapy (ref= Timepoint 1) | -1.068 (-2.99, 0.85)  p= 0.276 | -0.232 (-2.16, 1.7)  p= 0.814 | -3.74 (-6.62, -0.86)  p= 0.012* | -2.202 (-3.49, -0.92)  p= 0.001* | 1.363 (-0.23, 2.95)  p= 0.095 | 0.072 (-0.01, 0.15)  p= 0.07 |
| Stable (ref= Timepoint 1) | -0.122 (-1.94, 1.7)  p= 0.896 | 0.495 (-2.38, 3.37)  p= 0.736 | -5.332 (-9.52, -1.15)  p= 0.014* | -1.643 (-2.84, -0.45)  p= 0.008* | 1.547 (-0.85, 3.95)  p= 0.208 | 0.056 (-0.06, 0.17)  p= 0.353 |
|  |  |  |  |  |  |  |
| Chronological Age | 0.009 (-0.12, 0.14)  p= 0.892 | 0.01 (-0.08, 0.1)  p= 0.842 | -0.024 (-0.17, 0.12)  p= 0.747 | 0.009 (-0.08, 0.09)  p= 0.84 | -0.057 (-0.14, 0.03)  p= 0.178 | -0.003 (-0.01, 0)  p= 0.081^ |
| Chinese Descent | -1.809 (-3.58, -0.04)  p= 0.046* | -1.219 (-2.52, 0.08)  p= 0.069^ | -2.306 (-4.34, -0.27)  p= 0.027* | -0.789 (-1.95, 0.37)  p= 0.186 | -0.782 (-1.93, 0.37)  p= 0.184 | -0.037 (-0.09, 0.02)  p= 0.179 |
| Male Sex | 2.704 (1.25, 4.16)  p< 0.0001* | 2.148 (1.09, 3.21)  p< 0.0001* | -0.775 (-2.5, 0.96)  p= 0.381 | 0.191 (-0.79, 1.18)  p= 0.704 | 1.107 (0.15, 2.07)  p= 0.025* | 0.007 (-0.04, 0.05)  p= 0.762 |
| College Degree | -1.472 (-2.95, 0.01)  p= 0.053^ | -1.728 (-2.8, -0.65)  p= 0.002* | -2.254 (-3.95, -0.55)  p= 0.01* | -0.612 (-1.59, 0.37)  p= 0.222 | -1.933 (-2.86, -1)  p< 0.0001* | -0.066 (-0.11, -0.02)  p= 0.004* |
| Doxorubicin Exposure | -1.775 (-3.51, -0.04)  p= 0.047* | -0.39 (-1.73, 0.95)  p= 0.571 | 1.317 (-0.8, 3.43)  p= 0.224 | -0.386 (-1.58, 0.8)  p= 0.526 | 1.018 (-0.16, 2.2)  p= 0.093^ | 0.068 (0.01, 0.12)  p= 0.016* |
| Platinum Exposure | 0.357 (-1.16, 1.88)  p= 0.646 | 0.505 (-0.74, 1.75)  p= 0.428 | -1.967 (-3.95, 0.02)  p= 0.054^ | -0.208 (-1.29, 0.87)  p= 0.706 | -0.18 (-1.31, 0.95)  p= 0.756 | -0.03 (-0.08, 0.02)  p= 0.265 |
| Taxanes Exposure | -1.291 (-3.04, 0.45)  p= 0.149 | -1.54 (-2.87, -0.21)  p= 0.024* | -0.087 (-2.22, 2.04)  p= 0.936 | -0.315 (-1.51, 0.88)  p= 0.605 | -1.354 (-2.53, -0.18)  p= 0.026* | -0.054 (-0.11, 0)  p= 0.061^ |
| Radiation Exposure | 0.493 (-1.06, 2.04)  p= 0.534 | 2.096 (0.92, 3.27)  p= 0.001* | 3.162 (1.29, 5.03)  p= 0.001* | -0.051 (-1.12, 1.02)  p= 0.925 | 0.711 (-0.37, 1.79)  p= 0.199 | 0.016 (-0.04, 0.07)  p= 0.551 |
| Days Removed | 0.001 (0, 0)  p= 0.763 | -0.006 (-0.01, 0)  p= 0.019* | -0.011 (-0.02, 0)  p= 0.007* | -0.002 (-0.01, 0)  p= 0.324 | -0.006 (-0.01, 0)  p= 0.048* | 0 (0, 0)  p= 0.042* |
| PC1 | 0.004 (0, 0.01)  p= 1e-05* | 0 (0, 0)  p= 0.75574 | -0.003 (-0.01, 0)  p= 0.00607* | 0.002 (0, 0)  p= 0.00019* | 0 (0, 0)  p= 0.66527 | 0 (0, 0)  p= 0.24974 |
| PC2 | -0.003 (-0.01, 0)  p= 0.02718* | 0.003 (0, 0)  p= 0.002* | 0.006 (0, 0.01)  p= 4e-05* | 0 (0, 0)  p= 0.57826 | 0.003 (0, 0)  p= 2e-05* | 0 (0, 0)  p= 0.02434* |
| PC3 | -0.002 (-0.01, 0)  p= 0.40667 | -0.006 (-0.01, 0)  p= 1e-05* | -0.015 (-0.02, -0.01)  p< 0.0001* | -0.002 (0, 0)  p= 0.1271 | -0.005 (-0.01, 0)  p= 6e-05* | 0 (0, 0)  p= 0.00055* |
| PC4 | -0.008 (-0.01, 0)  p= 0.00074* | -0.006 (-0.01, 0)  p= 0.00031* | -0.005 (-0.01, 0)  p= 0.04777* | -0.003 (-0.01, 0)  p= 0.02303* | 0.001 (0, 0)  p= 0.55436 | 0 (0, 0)  p= 0.42655 |
| **Selected**  **Confounders** | Chinese Descent +  Male Sex +  College Degree +  Doxorubicin Exposure+  PC1 +  PC2 +  PC4 | Chinese Descent +  Male Sex +  College Degree +  Taxanes Exposure +  RadiationExposure+  Days Removed +  PC2 +  PC3 +  PC4 | Chinese Descent +  College Degree +  Platinum Exposure+  RadiationExposur+  Days Removed +  PC1 +  PC2 +  PC3 +  PC4 | PC1 +  PC4 | Male Sex +  College Degree +  Doxorubicin Exposure +  Taxanes Exposure +  Days Removed +  PC2 +  PC3 | Chronological Age +  College Degree +  Doxorubicin Exposure +  Taxanes Exposure +  Days Removed +  PC2 +  PC3 |

***PC- Principle component**

**Supplemental Table 3: Relationship of Epigenetic Ageing with Circulating Brain Derived Neurotrophic Factor Levels of Cancer Patients**

The first six rows are reported results specific to epigenetic ageing measure evaluated as a predictor in multivariable models, with rows below the black line representing univariable evaluations conducted to identify relevant confounders. At the bottom of the table, the selected confounders are listed for convenience. Each entry is reported as regression coefficients with corresponding 95% confidence intervals (Coefficient (95% CI)), for regression analyses conduced predicting circulating BDNF levels. P-values are reported beneath, with (*) indicators for values less than 0.05, and (^) symbols for values less than 0.1.

| **Feature** | **Circulating BDNF Levels** |
| --- | --- |
| *Horvath Age* | 0.029 (0, 0.05)  p= 0.024* |
| *Hannum Age* | 0.017 (-0.02, 0.05)  p= 0.341 |
| *PhenoAge* | -0.007 (-0.03, 0.02)  p= 0.562 |
| *Horvath2 Age* | 0.043 (0, 0.08)  p= 0.028* |
| *GrimAge* | -0.041 (-0.08, 0)  p= 0.028* |
| *DunedinPACE* | -0.717 (-1.48, 0.05)  p= 0.067 |
|  |  |
| *Chronological Age* | 0.007 (-0.014, 0.029)  p= 0.50045 |
| *Chinese Descent* | -0.223 (-0.529, 0.083)  p= 0.1554 |
| *Male Sex* | -0.064 (-0.32, 0.192)  p= 0.62386 |
| *College Degree* | 0.128 (-0.128, 0.384)  p= 0.32843 |
| *Doxorubicin Exposure* | -0.021 (-0.33, 0.289)  p= 0.89677 |
| *Platinum Exposure* | -0.211 (-0.463, 0.04)  p= 0.10163 |
| *Taxanes Exposure* | 0.168 (-0.14, 0.476)  p= 0.28723 |
| *Radiation Exposure* | 0.003 (-0.268, 0.274)  p= 0.98267 |
| *Active Chemotherapy*  *(Ref= Timepoint 1)* | -0.147 (-0.499, 0.204)  p= 0.44922 |
| *Recent Chemotherapy*  *(Ref= Timepoint 1)* | -0.122 (-0.471, 0.226)  p= 0.52207 |
| *Stable (Ref=Timepoint 1)* | -0.271 (-0.596, 0.054)  p= 0.16283 |
| *Days Removed* | -0.001 (-0.002, 0)  p= 0.01318* |
| *PC1* | 0 (0, 0)  p= 0.003* |
| *PC2* | 0 (0, 0)  p= 0.847 |
| *PC3* | -0.001 (0, 0)  p= 0.035* |
| *PC4* | 0 (0, 0)  p= 0.319 |
| **Selected Confounders** | Days Removed +  PC1 +  PC3 |

***PC- Principle component**

**Supplemental Table 4: Relationship of Epigenetic Ageing with Cognitive Function Outcomes in Cancer Patients**

The first six rows are reported results specific to epigenetic ageing measure evaluated as a predictor in multivariable models, with rows below the black line representing univariable evaluations conducted to identify relevant confounders. At the bottom of the table, the selected confounders are listed for convenience. Each entry is reported as regression coefficients with corresponding 95% confidence intervals (Coefficient (95% CI)), for regression analyses conduced predicting each cognitive function outcome represented in each column. P-values are reported beneath, with (*) indicators for values less than 0.05, and (^) symbols for values less than 0.1.

| **Feature** | **Fact-Cog Score** | **Subjective Impairment** | **Multi-Task RCI** | **Memory RCI** | **Response RCI** | **Executive Function RCI** | **Attention RCI** | **Objective Impairment** |
| --- | --- | --- | --- | --- | --- | --- | --- | --- |
| *Horvath Age* | 0.157 (-0.4, 0.72)  p= 0.583 | 0.016 (-0.11, 0.15)  p= 0.811 | -0.003 (-0.05, 0.05)  p= 0.898 | 0.024 (-0.03, 0.08)  p= 0.372 | -0.004 (-0.05, 0.04)  p= 0.855 | 0.014 (-0.02, 0.05)  p= 0.474 | 0.041 (0, 0.08)  p= 0.062 | -0.019 (-0.12, 0.08)  p= 0.723 |
| *Hannum Age* | -0.589 (-1.36, 0.19)  p= 0.14 | 0.198 (0.01, 0.38)  p= 0.037* | 0.028 (-0.04, 0.09)  p= 0.388 | -0.082 (-0.15, -0.01)  p= 0.021* | 0.017 (-0.04, 0.07)  p= 0.532 | 0.027 (-0.02, 0.07)  p= 0.256 | 0.01 (-0.05, 0.07)  p= 0.737 | 0.096 (-0.03, 0.22)  p= 0.146 |
| *PhenAge* | -0.557 (-1.01, -0.11)  p= 0.017* | 0.022 (-0.08, 0.12)  p= 0.675 | -0.025 (-0.06, 0.01)  p= 0.188 | -0.044 (-0.08, -0.01)  p= 0.024* | -0.006 (-0.04, 0.02)  p= 0.717 | 0.006 (-0.02, 0.03)  p= 0.632 | -0.02 (-0.05, 0.01)  p= 0.243 | 0.079 (0, 0.15)  p= 0.038* |
| *Horvath2 Age* | 0.145 (-0.66, 0.95)  p= 0.726 | 0.171 (-0.02, 0.37)  p= 0.084 | -0.029 (-0.11, 0.05)  p= 0.461 | -0.026 (-0.1, 0.05)  p= 0.516 | 0.044 (-0.02, 0.11)  p= 0.202 | 0.009 (-0.05, 0.06)  p= 0.758 | 0.011 (-0.06, 0.08)  p= 0.754 | 0.02 (-0.14, 0.18)  p= 0.805 |
| *GrimAge* | 0.17 (-0.63, 0.97)  p= 0.676 | -0.064 (-0.25, 0.12)  p= 0.504 | -0.038 (-0.1, 0.03)  p= 0.239 | -0.017 (-0.09, 0.05)  p= 0.638 | -0.041 (-0.09, 0.01)  p= 0.119 | 0.028 (-0.02, 0.08)  p= 0.278 | -0.062 (-0.12, 0)  p= 0.05 | 0.145 (0.02, 0.27)  p= 0.026* |
| *DunedinPACE* | -4.823 (-21.55, 11.91)  p= 0.573 | -1.217 (-4.78, 2.34)  p= 0.503 | -0.387 (-1.72, 0.95)  p= 0.571 | -1.126 (-2.52, 0.26)  p= 0.116 | -0.478 (-1.53, 0.58)  p= 0.377 | 0.284 (-0.67, 1.24)  p= 0.561 | -0.725 (-2, 0.54)  p= 0.265 | 1.981 (-0.54, 4.5)  p= 0.124 |
|  |  |  |  |  |  |  |  |  |
| *Chronological Age* | 0.646 (0.05, 1.24)  p= 0.034* | -0.094 (-0.17, -0.02)  p= 0.011* | -0.033 (-0.07, 0.01)  p= 0.102 | -0.072 (-0.11, -0.03)  p< 0.0001* | -0.018 (-0.05, 0.02)  p= 0.307 | -0.051 (-0.08, -0.02)  p= 0.001* | -0.047 (-0.08, -0.01)  p= 0.009* | 0.074 (-0.03, 0.18)  p= 0.156 |
| *Chinese Descent* | 10.824 (2.35, 19.3)  p= 0.014* | -0.565 (-1.59, 0.46)  p= 0.278 | -0.045 (-0.62, 0.53)  p= 0.879 | -0.455 (-1.01, 0.1)  p= 0.113 | 0.176 (-0.31, 0.66)  p= 0.482 | 0.04 (-0.41, 0.49)  p= 0.862 | 0.12 (-0.39, 0.63)  p= 0.646 | -0.347 (-1.47, 0.77)  p= 0.544 |
| *Male Sex* | 14.016 (7.21, 20.82)  p< 0.0001* | -0.944 (-2.02, 0.13)  p= 0.084^ | -0.036 (-0.51, 0.44)  p= 0.883 | -0.395 (-0.86, 0.07)  p= 0.098^ | 0.117 (-0.29, 0.52)  p= 0.575 | -0.126 (-0.5, 0.25)  p= 0.512 | 0.079 (-0.34, 0.5)  p= 0.714 | 0.731 (-0.22, 1.69)  p= 0.134 |
| *College Degree* | -3.358 (-10.53, 3.81)  p= 0.36 | 0.242 (-0.7, 1.19)  p= 0.616 | 0.2 (-0.27, 0.68)  p= 0.41 | -0.023 (-0.49, 0.44)  p= 0.924 | -0.01 (-0.42, 0.4)  p= 0.96 | 0.334 (-0.04, 0.7)  p= 0.08^ | 0.153 (-0.27, 0.58)  p= 0.478 | -0.208 (-1.18, 0.76)  p= 0.675 |
| *Doxorubicin Exposure* | -14.172 (-21.18, -7.17)  p< 0.0001* | 1.322 (0.4, 2.24)  p= 0.005* | 0.498 (0.02, 0.98)  p= 0.045* | 0.763 (0.3, 1.23)  p= 0.002* | 0.31 (-0.11, 0.73)  p= 0.146 | 0.643 (0.27, 1.01)  p= 0.001* | 0.38 (-0.05, 0.81)  p= 0.087^ | -0.718 (-1.89, 0.46)  p= 0.23 |
| *Platinum Exposure* | 17.099 (10.56, 23.64)  p< 0.0001* | -1.268 (-2.18, -0.35)  p= 0.007* | -0.482 (-0.95, -0.01)  p= 0.046* | -0.92 (-1.36, -0.48)  p< 0.0001* | -0.177 (-0.58, 0.23)  p= 0.393 | -0.174 (-0.55, 0.2)  p= 0.364 | 0 (-0.42, 0.42)  p= 0.999 | 0.605 (-0.49, 1.7)  p= 0.278 |
| *Taxanes Exposure* | -5.129 (-12.51, 2.25)  p= 0.176 | 0.715 (-0.19, 1.62)  p= 0.122 | -0.168 (-0.66, 0.32)  p= 0.504 | 0.284 (-0.2, 0.76)  p= 0.249 | -0.293 (-0.71, 0.12)  p= 0.169 | 0.451 (0.07, 0.83)  p= 0.022* | -0.2 (-0.64, 0.24)  p= 0.372 | -0.092 (-1.13, 0.94)  p= 0.862 |
| *Radiation Exposure* | 6.611 (-0.11, 13.33)  p= 0.056^ | -0.405 (-1.28, 0.47)  p= 0.366 | -0.113 (-0.56, 0.34)  p= 0.623 | -0.472 (-0.91, -0.04)  p= 0.035* | 0.139 (-0.24, 0.52)  p= 0.478 | 0.281 (-0.07, 0.63)  p= 0.12 | -0.332 (-0.73, 0.06)  p= 0.102 | 0.347 (-0.61, 1.3)  p= 0.475 |
| *Recent Chemotherapy (Ref= Active Therapy)* | 4.826 (-3.77, 13.43)  p= 0.322 | 0.036 (-0.95, 1.03)  p= 0.943 | 0.422 (-0.15, 1)  p= 0.21 | 0.32 (-0.25, 0.89)  p= 0.318 | 0.381 (-0.11, 0.87)  p= 0.186 | 0.261 (-0.19, 0.72)  p= 0.311 | 0.022 (-0.48, 0.53)  p= 0.934 | -0.683 (-1.88, 0.52)  p= 0.264 |
| *Stable (Ref= Active Therapy)* | 9.013 (0.92, 17.11)  p= 0.081^ | -1.464 (-2.7, -0.22)  p= 0.021* | 0.306 (-0.24, 0.85)  p= 0.318 | 0.283 (-0.25, 0.82)  p= 0.346 | 0.401 (-0.06, 0.86)  p= 0.147 | 0.112 (-0.32, 0.54)  p= 0.629 | 0.487 (0.01, 0.96)  p= 0.102 | -0.588 (-1.68, 0.51)  p= 0.292 |
| *MFSI Score* | -0.79 (-0.91, -0.67)  p< 0.0001* | 0.056 (0.03, 0.08)  p< 0.0001* | 0.012 (0, 0.02)  p= 0.048* | 0.012 (0, 0.02)  p= 0.054^ | -0.013 (-0.02, 0)  p= 0.012* | 0.005 (-0.01, 0.01)  p= 0.357 | -0.005 (-0.02, 0.01)  p= 0.384 | 0.005 (-0.02, 0.03)  p= 0.696 |
| *RSCL Score* | -0.672 (-0.82, -0.53)  p< 0.0001* | 0.068 (0.04, 0.1)  p< 0.0001* | 0.009 (0, 0.02)  p= 0.162 | 0.014 (0, 0.03)  p= 0.024* | -0.017 (-0.03, -0.01)  p= 0.001* | 0.004 (-0.01, 0.01)  p= 0.385 | 0 (-0.01, 0.01)  p= 0.953 | -0.004 (-0.03, 0.02)  p= 0.78 |
| *Days Removed* | 0.012 (-0.01, 0.03)  p= 0.267 | -0.003 (-0.01, 0)  p= 0.052^ | 0.001 (0, 0)  p= 0.406 | 0.001 (0, 0)  p= 0.091^ | 0 (0, 0)  p= 0.828 | 0 (0, 0)  p= 0.72 | 0.002 (0, 0)  p= 0.006* | -0.001 (0, 0)  p= 0.47 |
| *PC1* | 0.007 (0, 0.02)  p= 0.118 | 0 (0, 0)  p= 0.736 | 0 (0, 0)  p= 0.342 | -0.001 (0, 0)  p= 0.082^ | -0.001 (0, 0)  p= 0.029* | 0 (0, 0)  p= 0.582 | 0 (0, 0)  p= 0.522 | 0.001 (0, 0)  p= 0.148 |
| *PC2* | 0.005 (-0.01, 0.02)  p= 0.393 | 0 (0, 0)  p= 0.776 | 0.001 (0, 0)  p= 0.114 | 0 (0, 0)  p= 0.782 | 0.001 (0, 0)  p= 0.11 | 0.001 (0, 0)  p= 0.028* | 0 (0, 0)  p= 0.378 | 0.001 (0, 0)  p= 0.469 |
| *PC3* | 0.008 (-0.01, 0.03)  p= 0.321 | -0.001 (0, 0)  p= 0.462 | 0 (0, 0)  p= 0.38 | 0.001 (0, 0)  p= 0.168 | 0 (0, 0)  p= 0.964 | 0 (0, 0)  p= 0.648 | 0.001 (0, 0)  p= 0.231 | -0.002 (0, 0)  p= 0.11 |
| *PC4* | 0.007 (-0.02, 0.03)  p= 0.557 | -0.002 (-0.01, 0)  p= 0.107 | 0.001 (0, 0)  p= 0.476 | -0.001 (0, 0)  p= 0.393 | -0.001 (0, 0)  p= 0.078^ | -0.001 (0, 0)  p= 0.01* | -0.001 (0, 0)  p= 0.12 | 0.001 (0, 0)  p= 0.712 |
| **Selected Confounders** | Chronological Age +  Chinese Descent +  Male Sex +  Doxorubicin Exposure +  Platinum Exposure +  Radiation Exposure +  Treatment Status +  MFSI Score +  RSCL Score | Chronological Age +  Male Sex +  Doxorubicin Exposure +  Platinum Exposure +  Treatment Status +  MFSI Score +  RSCL Score +  Days Removed | Doxorubicin Exposure +  Platinum Exposure +  MFSI Score | Chronological Age +  Male Sex +  Doxorubicin Exposure +  Platinum Exposure +  Radiation Exposure +  MFSI Score +  RSCL Score +  Days Removed +  PC1 | MFSI Score +  RSCL Score+  PC1 +  PC4 | Chronological Age +  College Degree +  Doxorubicin Exposure +  Taxanes Exposure +  PC2 +  PC4 | Chronological Age +  Doxorubicin Exposure +  Days Removed | none |

***PC- Principle component**

**Supplemental Table 5: Total Differentially Methylated Positions (DMPs), Regions (DMRs), and Enriched Pathways Related to Measured Cognitive Function Outcomes and Circulating Brain Derived Neuropathic Factor (BDNF) Levels**

Each column represents a different outcome evaluated for differential methylation and pathway analysis. Cumulative total differentially methylated positions, regions, and enriched pathways are reported relative to each outcome. Additionally, the total number of differentially methylated positions and regions are reported within selected pathways. Genomic inflation factor (λ) relative to each outcome is reported.

|  | **Category** | FACT-Cog Score  λ= 1.35 | Memory RCI  λ= 0.95 | Response RCI  λ= 1.08 | Attention RCI  λ= 1.02 | Executive Function RCI  λ= 0.81 | Multitask RCI  λ= 1.32 | Circulating BDNF  λ= 1.02 |
| --- | --- | --- | --- | --- | --- | --- | --- | --- |
| **DMPs** | *Total* | 246 | 1592 | 277 | 4 | 65 | 297 | 335 |
|  |  |  |  |  |  |  |  |  |
| **DMRs** | *Total* | 9 | 28 | 5 | 4 | 9 | 14 | 8 |
|  |  |  |  |  |  |  |  |  |
|  | *Total Enriched Pathays* | 43 | 632 | 75 | 0 | 0 | 83 | 28 |
| **Pathways** | *KEGG Pathways* | 13 | 21 | 0 | 0 | 0 | 0 | 0 |
|  | *GO:Biological Process* | 0 | 418 | 55 | 0 | 0 | 48 | 19 |
|  | *GO:Molecular Function* | 2 | 64 | 15 | 0 | 0 | 23 | 0 |
|  | *GO:Cellular Component* | 28 | 129 | 5 | 0 | 0 | 12 | 9 |

**Supplemental Table 6: Enriched Pathways Relative to Circulating BDNF Levels**

From left to right, the first column represents the Gene ontology class, followed by the pathway name, and the pathway ID. All pathways listed meet the suggestive enrichment criteria, those meeting the stringent threshold are indicated with (*) symbols. There were no enriched pathways with KEGG or GO Molecular Process Collections to Report. **Bolded** pathway names have direct relevance to neuronal structure and function.

|  | **Name** | **ID** |
| --- | --- | --- |
| **GO: Biological Processes** | ***regulation of neuron projection development*** | GO:0010975 |
|  | *cellular anatomical entity morphogenesis* | GO:0032989 |
|  | ***cell morphogenesis involved in neuron differentiation*** | GO:0048667 |
|  | ***neuron projection morphogenesis*** | GO:0048812 |
|  | *cell projection morphogenesis* | GO:0048858 |
|  | *plasma membrane bounded cell projection morphogenesis* | GO:0120039 |
|  | ***generation of neurons*** | GO:0048699 |
|  | ***neurogenesis*** | GO:0022008 |
|  | *regulation of plasma membrane bounded cell projection organization* | GO:0120035 |
|  | *cell-cell signaling* | GO:0007267 |
|  | ***neuron differentiation*** | GO:0030182 |
|  | ***regulation of axon extension*** | GO:0030516 |
|  | ***neuron projection development*** | GO:0031175 |
|  | *regulation of cell projection organization* | GO:0031344 |
|  | *neuromuscular process controlling balance* | GO:0050885 |
|  | *regulation of nervous system development* | GO:0051960 |
|  | ***synaptic signaling*** | GO:0099536 |
|  | *cell morphogenesis* | GO:0000902 |
|  | *forebrain development* | GO:0030900 |
| **GO: Cellular Components** | ***Synapse*** | GO:0045202 |
|  | ***neuron projection*** | GO:0043005 |
|  | *myofibril* | GO:0030016 |
|  | *contractile fiber* | GO:0043292 |
|  | *glutamatergic synapse* | GO:0098978 |
|  | *sarcomere* | GO:0030017 |
|  | *Z disc* | GO:0030018 |
|  | *anchoring junction* | GO:0070161 |
|  | ***presynapse*** | GO:0098793 |

**Supplemental Table 7: Differentially Methylated Positions and Regions Associated with Brain Derived Neuropathic Factor Pathways**

Differentially methylated positions (DMPs) and regions (DMRs) within selected Gene Ontology and KEGG pathways are shown relative to each evaluated outcome. In the left column, the pathway’s descriptive name is listed first, followed its associated pathway ID (GO or KEGG), and the total number of genes within the pathway(n= gene count). In the right column, the differentially methylated positions and regions mapped to genes within the selected pathways relative to evaluated outcomes are listed

**Differentially methylated positions** (shown in **black**) are listed in the following format:Outcome, HGNC gene symbol, (Illumina probe ID, chromosome, base-pair position)

**Differentially methylated regions** (shown in **blue**) are listed as: Outcome, HGNC gene symbol, (chromosome, total probes, base-pair range)

| **Pathway** | **Implicated Methylation Sites and Regions** |
| --- | --- |
| *Neurotrophin Signaling Pathway*  **HSA:04722**  n= 126 genes | *FactCog Score*, **PIK3CD** (cg26573321, chr 1, pos= 9711663)  *FactCog Score*, **RPS6KA2** ( cg19231371, chr 6, pos= 167196254)  *Memory RCI*, **ATF4** (cg07080428, chr 22, pos= 39915234)  *Memory RCI*, **CAMK2A** (cg08631650, chr5, pos= 149669813)  *Memory RCI*, **GAB1** (cg01601573, chr4, pos= 144256835)  *Memory RCI*, **SH2B3** (cg01831767, chr12, pos= 111874281)  *Response RCI*, **SH2B2**  (cg01723606, chr7, pos= 101944275;  cg07512361, chr7, pos= 101944430)  *Executive Function RCI*, **RAP1A** (cg24160066, chr1, pos= 112161864)  *Executive Function RCI*, **RAP1A (chr 1, 3 total probes,**  pos= 112161618- 112161865)  *Multi-Task RCI*, **HRAS** (cg05798318, chr11, pos= 536758),  *Multi-Task RCI*, **SHC3** ( cg10242971, chr9, pos= 91692850) |
| *Neurotrophin Signaling Pathway*  **GO:0038179**  n= 40 genes | FactCog Score, **ZFYVE27** (cg02094827, chr10, pos= 99496515)  *Executive Function RCI*, **RAP1A** (cg24160066, chr1, pos= 112161864)  *Executive Function RCI*, **RAP1A (chr 1, 3 total probes,**  pos= 112161618- 112161865)  *Circulating BDNF,* **ZFYVE27 (cg02093062, chr10, pos=** 99496557) |
| *Neurotrophin Production*  **GO:0032898**  n= 5 genes | *FactCog Score*, **NPY** (cg01656438, chr7, pos= 24329741)  *Memory RCI*, **ADORA1**  (cg00256767, chr1, 203096899;  cg04822851, chr1, 203095988;  cg07232945, chr1, 203096570;  cg12794758, chr1, 203097234;  cg19315653, chr1, 203096230;  cg27480064, chr1, 203097247)  Memory RCI, **ADORA1 (chr 1, 6 total probes, pos=**  203096153- 203096684**)**  *Circulating BDNF*, **PCSK6** (cg15587362, chr15, pos= 101986985) |
| *Neurotrophin TRK Receptor Signaling Pathway*  **GO:0048011**  N= 26 genes | FactCog Score, **ZFYVE27** (cg02094827, chr10, pos= 99496515)  *Circulating BDNF,* **ZFYVE27 (cg02093062, chr10, pos=** 99496557) |
| **Neurotrophin Receptor Binding**  (GO:0005165)  n= 12 genes | *Response RCI*, **EFNA5 (**cg20740133**,** chr5, pos= 106792666) |
| **Neurotrophin TRK Receptor Binding**  (GO:0005167)  n= 6 genes | *Response RCI*, **EFNA5 (**cg20740133, chr5, pos= 106792666) |
| N**eurotrophin Binding**  (GO:0043121)  N= 10 genes | *Circulating BDNF*, **PCSK6** (cg15587362, chr15, pos= 101986985) |
| **Cellular Response to Brain Derived Neuropathic Factor Stimulus**  (GO:1990416)  n= 10 genes | *Memory RCI,* **MAPT***(* *cg15617032, chr17, pos=*  *44020778)* |
| *Positive Regulation of Neurotrophin TRK Receptor Signaling Pathway*  **(GO:0051388)**  n= 5 genes |  |
| *Regulation of Neurotrophin TRK Receptor Signaling Pathway*  **(** **GO:0051386)**  n= 11 genes |  |
| *Neurotrophin Receptor Activity*  **(GO:0005030)**  n= 5 genes |  |
| *Brain Derived Neuropathic Factor*  *Signaling Pathway*  **(GO:0031547)**  n= 6 genes |  |
