# Supplementary material for: Epigenetic age acceleration and neurotrophin signaling pathways in cancer-related cognitive impairment: a longitudinal, prospective cohort study
Source: Front Aging. 2025 Dec 1;6:1667638. doi: 10.3389/fragi.2025.1667638 (PMC12702849; doi:10.3389/fragi.2025.1667638)
Supplement: Supplementary file 2 [file Table2.docx]

**Supplemental Methods:**

**Section 1:Management of Measured Symptom Data**

*Cambridge Neuropsychological Test Automated Battery (CANTAB) tests*

Objective cognitive function measures were captured with Cambridge Neuropsychological Test Automated Battery (CANTAB) tests, evaluating cognitive domains of memory, response speed, executive function, attention, and multi-tasking.^1,2^ These assessments are performed on a tablet, where the software has patients perform tasks that measure their cognitive capacities specific to each domain. Reliable change indices (RCI) for each cognitive domain were calculated by subtracting the raw scores, divided by the standard error of difference estimated from the non-cancer group in order to account for practice effects.^3^All scores have been adjusted such that a positive RCI indicates an improvement for the measured domain from baseline, while a negative RCI represents a decline from baseline. Post-baseline reliable change indices were utilized in subsequent analyses from timepoints 2 through 5. Additionally, a separate “clinically significant objective cognitive impairment” outcome was evaluated, defined as a RCI < − 1.96 (< 5% probability of deteriorating by chance).^4^

*The Functional Assessment of Cancer Therapy-Cognitive Function version 3 (FACT-Cog)*

Subjective cognition function was captured with The Functional Assessment of Cancer Therapy-Cognitive Function version 3 (FACT-Cog). This questionnaire evaluates four sub-categories including perceived cognitive impairments, perceived cognitive abilities, comments from others, and impact on quality of life. Responses are in the form of a Likert scale for 37 total items, and are subsequently summed to create the cumulative total score (0 to 148).^5,6^ Higher scores indicate better self-perceived cognitive function.“Clinically significant subjective cognitive impairment” was defined as measured total Fact-Cog scores having a ≥ 10.6-point decline relative to baseline scores.^7^

*Multidimensional Fatigue Symptom Inventory-Short Form*

Fatigue symptoms were characterized utilizing the Multidimensional Fatigue Symptom Inventory-Short Form (MFSI-SF).^8^ MFSI-SF consists of a variety of questions representing 5 distinct domains of fatigue (general, physical, emotional, mental, vigor) , each scored from 0 to 4 representing symptom severity. Cumulative fatigue score is obtained by summing all of the scores except for questions related to vigor, which are subsequently subtracted from the total score. Total fatigue score ranges from -24 to 96, with higher scores indicating more fatigue symptoms and higher severity.

*Rotterdam Symptom Checklist*

Symptoms of psychological distress were captured utilizing with the with the psychological distress domain of Rotterdam Symptom Checklist (RSCL-PD). RSCL evaluates symptoms reported by cancer patients and covering 4 distinct domains (physical symptom distress, psychological distress, activity level, global life quality) with several questions graded on a 4 point likert scale.^9^ Cumulative psychological symptom score is obtained by transforming the summed likert scale values to a 100-point scale, with higher values indicating a higher or worsening symptom burden.

**Section 2: Management of DNA Methylation Measurements**

*DNA Methylation Measurements*

Buffy coat samples collected from blood draws were utilized for DNA methylation measurements, performed using the Illumina Infinium® Methylation EPIC Array platform. The methylation raw data was initially processed in the R platform, utilizing various R packages (minfi, waterMelon).^10,11^ Samples were included if they met the following quality control measures: average P value detection level <0.01 across all probes, correct methylation predicted biological sex, and a bisulfate conversion rate of 90% or higher.

For purposes of utilizing various epigenetic age metrics DNA methylation levels were normalized utilizing Noob normalization methods and converted to beta values as recommended for online calculators (<https://dnamage.genetics.ucla.edu/>) and R packages to obtain DNA epigenetic ageing metrics.^12-14^ Epigenetic ageing metrics considered included Horvath, Hannum, PhenoAge, Horvath Skin and Blood (Horvath2), GrimAge, and DunedinPace.^12,13,15-18^ The residuals of epigenetic age on chronological age were utilized in the analyses with all metrics except for DunedinPace (Supplemental Table 1). Ageing was characterized as “Accelerated” when residual values were greater than 0, suggesting increased ageing relative to expected given a persons chronological age within the population. For DunedinPACE, measures greater than 1indicated increased age acceleration relative to expected.

For subsequent analyses, methylation data were pre-processed utilizing the functional normalization method from minfi in R to account for batch effects in addition to background noise.^10^ Probes were removed from consideration if they were mapped to X and Y chromosomes or known to be cross reactive.^19-21^ Furthermore, probes having mean detection p-values greater than 0.01, minor allele frequencies greater than 5%, or average bead counts less than 3 across all samples were removed from consideration. To consider DNA methylation sits with meaningful biological variation, probes having a measured standard deviation of beta values across all samples less than 0.01 across all samples were not considered in differential methylation analyses.^22,23^ Genomic associations for each probe, including reference genes associated with each probe and larger genomic associations, were pulled from the EPIC illumina manifest file.^19,24^ Principle components were determined using irlba package in R, utilizing M-values matrices as input, where resulting PCs were centered and scaled.^25^ The top principle components were selected, explaining 80% of total observed variance in the data set utilized in the analysis.

*Sites Utilized for Differential Methylation Analysis*

All samples collected met pre-defined quality control thresholds for methylation measurements. Within each micro-array, 866,238 probes measured DNA methylation at distinct sites. After removing cross reactive probes, probes mapped to X and Y chromosomes, and probes not meeting pre-defined quality control measures 762,224 probes were considering for identification of DMPs. Amongst eligible probes, 566,354 met the pre-defined variance threshold to be considered in DMP evaluations.

**Section 3:Execution and Rationale of Statistical Analysis Approaches**

*Linear Mixed Models*

To account for the underlying trends of our longitudinal data structure, we implemented linear mixed effects linear regression models in several different analysis steps. Random effects were accounted for in multiple ways, depending on the analysis conducted. For analyses considering epigenetic age as an outcome, random effects were characterized utilizing the timepoint the measurements were collected. For analyses considering cognitive outcomes, random effects were accounted for utilizing a combination of treatment trajectory labels (prior to anticancer therapy, active cancer therapy, recent cancer therapy, stable (6+months removed from therapy) and timepoints to create distinct groupings. In our execution of this analysis, we attempted to utilized patient ids to account for random effects at an individual level. Due to limited measurements for several patients, the majority having only 1 or 2 post-baseline measurements, unstable estimates of variance made regression results unstable and inconclusive. Statistical modeling was performed in the R-programing platform, utilizing a combination of dplyr and tidyr to format data and nlme package to execute linear mixed models.^26,27^

*Identification of Differentially Methylated Positions*

For identification of differentially methylated positions, linear mixed effects models were implemented as described above. For each prospective outcome, M-values were utilized as the primary predictor of interest alongside relevant confounders of each prospective outcome. M-values were selected as opposed to beta values, as researchers have suggested that they have better statistical properties to achieve meaningful regression results.^28^ Each M-value was evaluated in separate regression analyses, repeated for every methylation site considered. To account for repeated analyses, Benjamini-Hochberg (BH) corrected p-values (q-values) were utilized assuming a false discovery rate of 5% correcting Wald-statistic p-values obtained from independently iterated regression analyses. This analysis conducted using R-program in combination with parallel computing on a remote server to manage heavy computational burden.^29^

To assess potential inflation of test statistics, we calculated the genomic inflation factor relative to each outcome assessed. The genomic inflation factor was calculated as the median of all chi-squared test statistics derived from independent regression analyses, divided by the expected median of the chi-squared distribution. Genomic inflation factors ~1 suggest an expected distribution of significance values, while those higher suggest unaccounted for confounding or systemic bias.^30,31^

*Identification of Differentially Methylated Regions*

The “Combp” algorithm was implemented to identify differentially methylated regions relative to outcomes of interest from the EnMix package in the R-platform. This algorithm utilizes Wald statistic p-values associated with methylation sites from DMP evaluations, along with their associated genomic coordinates, as inputs.^32^ Combp uses a sliding window spatial smoothing approach to identify differentially methylated regions (DMRs), by grouping nearby significant CpG sites, correcting for spatial autocorrelation, and adjusting for multiple testing across regions. A seed value to 0.001 was selected as a starting point for potential DMRs, with a 1,000 base pair cut-off for genomic autocorrelation. Significant DMRs had sidak corrected p-values of less 0.05 and 3 or more DNAm sites associated with the region. Furthermore, regression coefficients of all implicated sites within a DMR were required to be the same with resepect to directionality (all positive or all negative), to ensure DNAm relationships for a prospective cluster were consistent. Association of DMRs with mapped genes and promoter regions were based on mapping of individual sites within them; DMRs containing DMPs meeting the stringent significance criteria (q<0.05) were highlighted.

*Identification of Significantly Enriched Pathways*

Gene set enrichment analysis was conducted using the missMethyl package, specifically with the “gometh” function.^33^ Pathway enrichment was evaluated using the hypergeometric test, which assesses whether the number of significant genes associated with a given pathway is greater than expected by chance. To maintain simplicity and interpretability, probe- and gene-level weighting adjustments were not applied. Given our limited sample size, we lacked sufficient power to detect consistent signal across all probes associated with a gene. Four distinct libraries of broader genomic pathways were considered including the Kyoto Encyclopedia of Genes and Genome (KEGG) and Gene Ontology (GO) libraries related to cellular components, biological processes, and molecular function. Pathways considered within each of these sub-categories were limited to a range of 10-2000 genes. After enrichment analysis, BH correction of p-values from hypergeometric tests was performed within each pathway sub-category described above.

**Supplemental Methods References:**

**Sources:**

1. Chan A, Cheng I, Wang C, et al. Cognitive impairment in adolescent and young adult cancer patients: Pre-treatment findings of a longitudinal study. *Cancer Med*. Feb 2023;12(4):4821-4831. doi:10.1002/cam4.5295

2. Wefel JS, Vardy J, Ahles T, Schagen SB. International Cognition and Cancer Task Force recommendations to harmonise studies of cognitive function in patients with cancer. *Lancet Oncol*. Jul 2011;12(7):703-8. doi:10.1016/s1470-2045(10)70294-1

3. Maassen GH, Bossema E, Brand N. Reliable change and practice effects: outcomes of various indices compared. *J Clin Exp Neuropsychol*. Apr 2009;31(3):339-52. doi:10.1080/13803390802169059

4. Jacobson NS, Truax P. Clinical significance: a statistical approach to defining meaningful change in psychotherapy research. *J Consult Clin Psychol*. Feb 1991;59(1):12-9. doi:10.1037//0022-006x.59.1.12

5. Cheung YT, Lim SR, Shwe M, Tan YP, Chan A. Psychometric properties and measurement equivalence of the English and Chinese versions of the functional assessment of cancer therapy-cognitive in Asian patients with breast cancer. *Value Health*. Sep-Oct 2013;16(6):1001-13. doi:10.1016/j.jval.2013.06.017

6. Cella DF, Tulsky DS, Gray G, et al. The Functional Assessment of Cancer Therapy scale: development and validation of the general measure. *J Clin Oncol*. Mar 1993;11(3):570-9. doi:10.1200/jco.1993.11.3.570

7. Cheung YT, Foo YL, Shwe M, et al. Minimal clinically important difference (MCID) for the functional assessment of cancer therapy: cognitive function (FACT-Cog) in breast cancer patients. *J Clin Epidemiol*. Jul 2014;67(7):811-20. doi:10.1016/j.jclinepi.2013.12.011

8. Chan A, Lew C, Wang XJ, et al. Psychometric properties and measurement equivalence of the Multidimensional Fatigue Syndrome Inventory- Short Form (MFSI-SF) amongst breast cancer and lymphoma patients in Singapore. *Health Qual Life Outcomes*. Jan 19 2018;16(1):20. doi:10.1186/s12955-018-0846-6

9. Chan A, Poon E, Goh WL, et al. Assessment of psychological distress among Asian adolescents and young adults (AYA) cancer patients using the distress thermometer: a prospective, longitudinal study. *Support Care Cancer*. Sep 2018;26(9):3257-3266. doi:10.1007/s00520-018-4189-y

10. Aryee MJ, Jaffe AE, Corrada-Bravo H, et al. Minfi: a flexible and comprehensive Bioconductor package for the analysis of Infinium DNA methylation microarrays. *Bioinformatics*. May 15 2014;30(10):1363-9. doi:10.1093/bioinformatics/btu049

11. Pidsley R, CC YW, Volta M, Lunnon K, Mill J, Schalkwyk LC. A data-driven approach to preprocessing Illumina 450K methylation array data. *BMC Genomics*. May 1 2013;14:293. doi:10.1186/1471-2164-14-293

12. Horvath S. DNA methylation age of human tissues and cell types. *Genome Biol*. 2013;14(10):R115. doi:10.1186/gb-2013-14-10-r115

13. Belsky DW, Caspi A, Corcoran DL, et al. DunedinPACE, a DNA methylation biomarker of the pace of aging. *Elife*. Jan 14 2022;11doi:10.7554/eLife.73420

14. Triche TJ, Jr., Weisenberger DJ, Van Den Berg D, Laird PW, Siegmund KD. Low-level processing of Illumina Infinium DNA Methylation BeadArrays. *Nucleic Acids Res*. Apr 2013;41(7):e90. doi:10.1093/nar/gkt090

15. Hannum G, Guinney J, Zhao L, et al. Genome-wide methylation profiles reveal quantitative views of human aging rates. *Mol Cell*. Jan 24 2013;49(2):359-367. doi:10.1016/j.molcel.2012.10.016

16. Lu AT, Quach A, Wilson JG, et al. DNA methylation GrimAge strongly predicts lifespan and healthspan. *Aging (Albany NY)*. Jan 21 2019;11(2):303-327. doi:10.18632/aging.101684

17. Levine ME, Lu AT, Quach A, et al. An epigenetic biomarker of aging for lifespan and healthspan. *Aging (Albany NY)*. Apr 18 2018;10(4):573-591. doi:10.18632/aging.101414

18. Horvath S, Oshima J, Martin GM, et al. Epigenetic clock for skin and blood cells applied to Hutchinson Gilford Progeria Syndrome and ex vivo studies. *Aging (Albany NY)*. Jul 26 2018;10(7):1758-1775. doi:10.18632/aging.101508

19. Pidsley R, Zotenko E, Peters TJ, et al. Critical evaluation of the Illumina MethylationEPIC BeadChip microarray for whole-genome DNA methylation profiling. *Genome Biol*. Oct 7 2016;17(1):208. doi:10.1186/s13059-016-1066-1

20. McCartney DL, Walker RM, Morris SW, McIntosh AM, Porteous DJ, Evans KL. Identification of polymorphic and off-target probe binding sites on the Illumina Infinium MethylationEPIC BeadChip. *Genom Data*. Sep 2016;9:22-4. doi:10.1016/j.gdata.2016.05.012

21. Chen M. max probes (GitHub Respository). <https://github.com/markgene/maxprobes>

22. Welsh H, Batalha C, Li W, et al. A systematic evaluation of normalization methods and probe replicability using infinium EPIC methylation data. *Clin Epigenetics*. Mar 11 2023;15(1):41. doi:10.1186/s13148-023-01459-z

23. Hu X, Logan JG, Kwon Y, et al. Multi-ancestry epigenome-wide analyses identify methylated sites associated with aortic augmentation index in TOPMed MESA. *Sci Rep*. Oct 17 2023;13(1):17680. doi:10.1038/s41598-023-44806-z

24. Hansen K. IlluminaHumanMethylationEPICanno.ilm10b2.hg19: Annotation for Illumina's EPIC methylation arrays. 2016.

25. Baglama J, Reichel L, Lewis BW. irlba: Fast Truncated Singular Value Decomposition and Principal Components Analysis for Large Dense and Sparse Matrices. R package version 2.3.5.1 ed2022.

26. Hadley Wickham RF, Lionel Henry, Kirill Müller. DPLYR: Overview. 1. <https://dplyr.tidyverse.org/>

27. *nlme: Linear and Nonlinear Mixed Effects Models*. 2025. <https://CRAN.R-project.org/package=nlme>

28. Du P, Zhang X, Huang CC, et al. Comparison of Beta-value and M-value methods for quantifying methylation levels by microarray analysis. *BMC Bioinformatics*. Nov 30 2010;11:587. doi:10.1186/1471-2105-11-587

29. Kuhn M. Building Predictive Models in R Using the caret Package.

30. Nabais MF, Laws SM, Lin T, et al. Meta-analysis of genome-wide DNA methylation identifies shared associations across neurodegenerative disorders. *Genome Biol*. Mar 26 2021;22(1):90. doi:10.1186/s13059-021-02275-5

31. Devlin B, Roeder K. Genomic control for association studies. *Biometrics*. Dec 1999;55(4):997-1004. doi:10.1111/j.0006-341x.1999.00997.x

32. Jia T, Chu C, Liu Y, et al. Epigenome-wide meta-analysis of blood DNA methylation and its association with subcortical volumes: findings from the ENIGMA Epigenetics Working Group. *Mol Psychiatry*. Aug 2021;26(8):3884-3895. doi:10.1038/s41380-019-0605-z

33. Phipson B, Maksimovic J, Oshlack A. missMethyl: an R package for analyzing data from Illumina's HumanMethylation450 platform. *Bioinformatics*. Jan 15 2016;32(2):286-8. doi:10.1093/bioinformatics/btv560
